# ISI_matsuda_ as a potential predictor of metabolic dysfunction-associated steatotic liver disease in patients with type 2 diabetes mellitus

**DOI:** 10.3389/fmed.2025.1623808

**Published:** 2025-08-29

**Authors:** Jing Liu, Yueqiu Wang, Xinghang Zhou, Zaixin Wen, Yu Chen, Yiqiong Sun, Shuaiying Su, Weiwei Lin, Ruiting Shen, Xiaoyu Sun, Hongru Li, Xia Yu, Mingchen Zhang

**Affiliations:** ^1^Cixi Biomedical Research Institute, Wenzhou Medical University, Wenzhou, Zhejiang, China; ^2^Department of Endocrinology and Metabolism, Ningbo No.2 Hospital, Ningbo, Zhejiang, China; ^3^College of Information Science and Engineering, Northeastern University, Shenyang, Liaoning, China; ^4^College of Medicine, Shaoxing University, Shaoxing, Zhejiang, China; ^5^College of Medicine, Zhejiang Chinese Medical University, Hangzhou, Zhejiang, China

**Keywords:** insulin resistance, Matsuda index, metabolic dysfunction associated steatotic liver disease, type 2 diabetes mellitus, liver fibrosis

## Abstract

**Background:**

This study aimed to determine the most efficacious insulin resistance (IR) indices to predict metabolic dysfunction-associated steatotic liver disease (MASLD) in patients with type 2 diabetes mellitus (T2DM).

**Methods:**

This cross-sectional study included 1,587 patients with T2DM. MASLD was defined by abdominal ultrasound findings. Liver fibrosis risk was assessed with FIB-4. All participants underwent a 100 g standard steamed bread meal test. We analyzed basal IR indices (HOMA-IR, QUICKI, IAI, Bennett ISI) and post-stimulation IR indices (ISI_matsuda_, ISI_0,120_) to explore their associations with MASLD and liver fibrosis.

**Results:**

Participants were categorized into four groups according to IR indices quartiles. Among post-stimulation IR indices, MASLD detection rates in ISI_matsuda_ Q1–Q4 groups were 65.7, 54.2, 37.0, and 22.2%, respectively. Logistic regression analysis revealed significantly increased odds ratios (ORs) for MASLD in ISI_matsuda_ Q1-Q3 groups compared to the Q4 group (OR = 3.63, 2.53, and 1.53, respectively; all *p* < 0.05). Similar results were observed across other IR indices (all *p* < 0.05). There were no statistically significant differences in the detection rates of liver fibrosis or the ORs among the quartile groups of the IR indices (all *p* > 0.05). ROC curve analysis showed that ISI_matsuda_ had superior predictive power for MASLD in patients with T2DM (AUC = 0.701). Based on these findings, a risk prediction model for MASLD in the T2DM population was constructed using age, body mass index (BMI), alanine aminotransferase (ALT), triglycerides (TG), and 2-h postprandial C-peptide (2 h CP).

**Conclusion:**

Among the IR indices, ISI_matsuda_ demonstrated the strongest correlation and highest predictive value for MASLD in T2DM.

## 1 Introduction

The prevalence of metabolic dysfunction-associated steatotic liver disease (MASLD) globally continues to rise, now exceeding 30%, with approximately 11% of cases progressing to liver fibrosis (1–3). Among those with type 2 diabetes mellitus (T2DM), the incidence of MASLD ranges from 50 to 80%, with 17 to 36% of patients experiencing progressive liver fibrosis (4–6).

Insulin resistance (IR) is recognized as a central pathophysiological mechanism in the development of both T2DM and MASLD (7). IR not only constitutes the fundamental pathogenic basis of T2DM but also persists throughout the entire course of the disease. IR promotes hepatic *de novo* lipogenesis (DNL) and enhances lipolysis, leading to increased influx of free fatty acids (FFA) into the liver, which drives the development and progression of steatotic liver disease (8). The progression from IR to “second hits” has become the widely accepted framework for the pathogenesis of fatty liver disease. Patients with diabetes frequently have coexisting fatty liver disease, which progresses more rapidly in this population. However, the status of IR, especially post-stimulation IR, in patients with both diabetes and fatty liver disease remains underexplored. As some antidiabetic agents can improve IR, accurately assessing IR in this high-risk population not only helps elucidate underlying pathophysiological mechanisms but also facilitates more precise therapeutic decision-making.

The hyperinsulinemic euglycemic clamp (HEC) technique is regarded as the gold standard for assessing IR; however, its clinical application is limited by its invasiveness, high cost, and complex procedure (9). Clinically, IR assessment indices are categorized into basal indices and post-stimulation indices. Basal indices encompass the homeostasis model assessment of insulin resistance (HOMA-IR), quantitative insulin sensitivity check index (QUICKI), Lee index (IAI), and Bennett insulin sensitivity index (Bennett ISI), while post-stimulation indices include the Matsuda index (ISI_matsuda_) and Gutt index (ISI_0,120_) (10, 11). Current research primarily focuses on fasting IR, particularly the association between HOMA-IR and hepatic steatosis, with relatively little attention paid to post-stimulation IR. Furthermore, the relationship between IR and the progression of liver fibrosis remains unclear. This study aims to investigate the relationship between IR indices and the risk of MASLD and liver fibrosis in patients with T2DM.

## 2 Methods

### 2.1 Subjects of study

**Figure 1:**
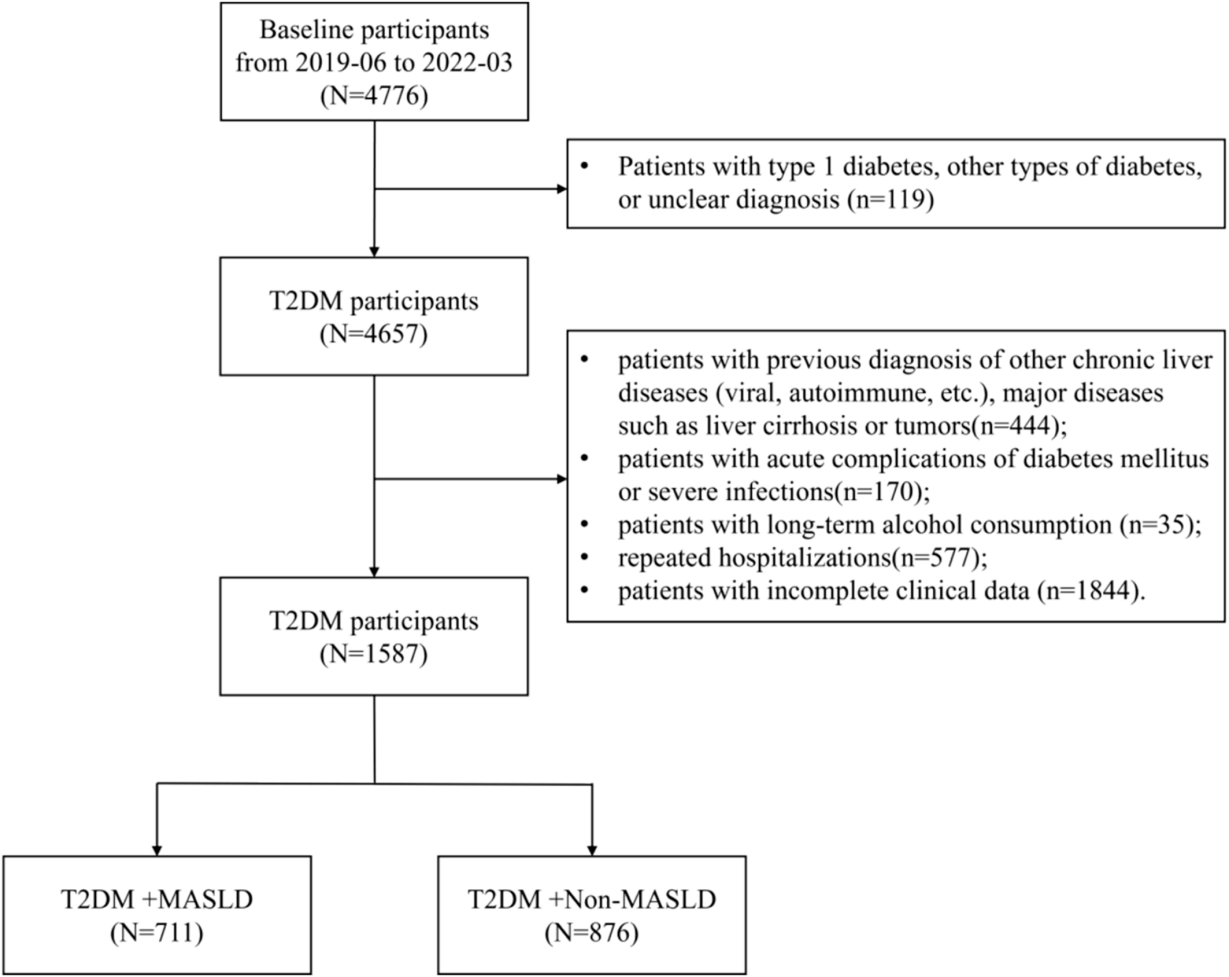
Flow diagram of patient selection.

This cross-sectional study adhered to the principles outlined in the Declaration of Helsinki and received approval from the Ethics Committee of Ningbo No. 2 Hospital (ethics approval number: YJ-NBET-KY-2022-130-01) and waived individual informed consent as only anonymized data were utilized. A total of 4,776 diabetic patients consecutively admitted to the Department of Endocrinology at Ningbo No. 2 Hospital between June 2019 and March 2022 were selected. Inclusion criteria included the following: (1) age ≥ 18 years; (2) diagnosis of diabetes in accordance with the 1999 WHO diagnostic criteria (12); (3) complete inpatient medical records. Exclusion criteria included the following: (1) type 1 diabetes, other types of diabetes, or unclear diagnosis (*n* = 119); (2) history of other chronic liver diseases (e.g., viral, autoimmune), liver cirrhosis, tumors, or other major diseases (*n* = 444); (3) acute diabetic complications or severe infections (*n* = 170); (4) history of long-term alcohol consumption (*n* = 35); (5) repeated hospitalizations (*n* = 577); (6) missing clinical data including BMI, fasting and 2-h postprandial plasma glucose and C-peptide (*n* = 1,844). Ultimately, 1,587 patients with T2DM were included in the study (Figure 1).

### 2.2 Data collection and processing

#### 2.2.1 General information

Clinical data were collected from electronic medical records, including age, gender, height, weight, diabetes history, and liver ultrasound results. The body mass index (BMI) was subsequently calculated. Serum biomarkers, such as total cholesterol (TC), triglycerides (TG), high-density lipoprotein cholesterol (HDL-C), low-density lipoprotein cholesterol (LDL-C), uric acid (UA), aspartate aminotransferase (AST), alanine aminotransferase (ALT), gamma-glutamyltransferase (*γ*-GGT), and glycated hemoglobin (HbA1c), were also measured.

#### 2.2.2 The 100 g standard steamed bread meal test

The carbohydrate content of a 100 g steamed bread meal is approximately equivalent to 75 g of glucose. Research conducted by the Chinese Islet Beta-Cell Function Collaborative Research Group demonstrated that the 100 g steamed bread meal test showed good reproducibility and tolerability for assessing *β*-cell function, in comparison to the oral glucose tolerance test (OGTT) (13). Consequently, we employed the 100 g steamed bread meal test to measure fasting plasma glucose (FPG), 2-h postprandial glucose (2 h PG), fasting C-peptide (FCP), and 2-h postprandial C-peptide (2 h CP). C-peptide levels were quantified using a magnetic microparticle chemiluminescence assay.

#### 2.2.3 Evaluation of IR

Due to the administration of insulin therapy to some patients during their hospital stay, C-peptide and insulin are co-secreted in equimolar ratios (14). Consequently, this study employed FPG and FCP measurements to calculate HOMA-IR, QUICKI, IAI, and Bennett ISI to assess basal IR. Additionally, fasting and 2-h post-meal blood glucose and C-peptide following the steamed bread meal test were used to compute ISI_matsuda_ and ISI_0,120_ to evaluate post-stimulation IR status. The formulas for these calculations are detailed in Supplementary Table S1.

#### 2.2.4 MASLD and liver fibrosis assessment criteria

The diagnosis of MASLD was determined through abdominal ultrasound findings indicating fatty liver or discharge diagnosis codes, such as K76.0. According to the American Association of Clinical Endocrinology (AACE) clinical practice guideline for the management of non-alcoholic fatty liver disease (NAFLD), the fibrosis risk in T2DM patients with MASLD was assessed using the non-invasive fibrosis-4 index (FIB-4) (15). FIB-4 < 1.3 indicated low risk, 1.3 ≤ FIB-4 ≤ 2.67 indicated intermediate risk, and FIB-4 > 2.67 indicated high risk.

The FIB-4 calculation formula was: FIB-4 = (Age (years) × AST (U/L))/(PLT (10^9^/L)) × (ALT (U/L)^1/2^).

### 2.3 Statistical analysis

Statistical analysis and graphical plotting were performed using SPSS version 26.0 (IBM Corp, NY, USA) and R software version 4.1.1 (R Foundation for Statistical Computing, Vienna, Austria). Categorical data were expressed as frequency (%), and group comparisons were conducted using the *χ*^2^ test. Continuous variables with a normal distribution were expressed as mean ± standard deviation ( $\bar{x}$ ± s), and comparisons between two groups were performed using an independent samples t-test. For multiple group comparisons, one-way analysis of variance (ANOVA) was applied. Non-normally distributed data were presented as M (P25, P75), and comparisons between two groups were performed using the Mann–Whitney U test, while comparisons among multiple groups were conducted using the Kruskal–Wallis H test.

The participants were categorized into four groups according to the quartiles of IR indices: Q1 (<25%), Q2 (25–50%), Q3 (50–75%), and Q4 (≥75%). Multicollinearity was assessed via variance inflation factors (VIF). After adjusting for potential confounders, logistic regression analysis was employed to examine the relationship between IR levels and the occurrence of MASLD and liver fibrosis. The diagnostic value of different IR indices for the onset of MASLD and liver fibrosis in patients with T2DM was assessed using receiver operating characteristic (ROC) curves.

Moreover, a predictive model for the risk of MASLD among the T2DM population was developed using logistic regression, incorporating age, BMI, ALT, and TG. The effectiveness of the model, before and after adding 2 h CP, was assessed using the concordance index (C-index), net reclassification improvement (NRI), and integrated discrimination improvement (IDI). Specifically, the NRI measures the extent to which the addition of a new variable improves the correct reclassification of individuals into clinically relevant risk categories, while the IDI reflects the improvement in the model’s ability to discriminate between cases and non-cases. The nomogram for the model was generated using the “rms” package. A *p*-value of <0.05 was considered statistically significant.

## 3 Results

This study encompassed 1,587 patients with T2DM, with an average age of 51.86 ± 13.07 years. Of these, 642 (40.45%) were female and 945 (59.55%) were male. A total of 711 (44.80%) patients with T2DM were diagnosed with MASLD, of which 271 (38.12%) were at intermediate risk of liver fibrosis and 46 (6.47%) were at high risk for liver fibrosis.

### 3.1 Comparison of clinical characteristics between T2DM patients with and without MASLD

Patients were divided into two groups based on the presence of MASLD: those with T2DM and MASLD (DM + MASLD group, *n* = 711) and those with T2DM but without MASLD (DM + Non-MASLD group, *n* = 876). Compared with the DM + Non-MASLD group, the DM + MASLD group was younger (55.07 vs. 60.66, *p* < 0.001) and had significantly higher levels of BMI (25.92 vs. 22.98), AST (24 vs. 20), ALT (27 vs. 18), *γ*-GGT (33 vs. 22), FPG (8.86 vs. 8.39), FCP (1.69 vs. 1.12), and 2 h CP (6.10 vs. 4.07) (all *p* < 0.01). Moreover, a higher proportion of the DM + MASLD group was overweight/obese (69.90%) compared with the DM + Non-MASLD (34.36%) group (*p* < 0.001). There were no significant differences in gender or HbA1c levels between the two groups (*p* > 0.05).

Compared with the DM + Non-MASLD group, in the fasting state, the DM + MASLD group showed significantly higher HOMA-IR (0.63 vs. 0.37) and lower values for QUICKI (0.20 vs. 0.21), IAI (0.07 vs. 0.12), and Bennett ISI (0.40 vs. 0.45) (all *p* < 0.001). Following a 100 g steamed bread meal stimulus, the DM + MASLD group demonstrated lower ISI_matsuda_ (1.14 vs. 1.82) and ISI_0,120_ (16.13 vs. 16.80) values (all *p* < 0.001). (Table 1).

**Table 1:** Clinical characteristics of T2DM patients with and without MASLD.

Variables	DM + MASLD (*n* = 711)	DM + Non-MASLD (*n* = 876)	χ^2^/t/Z	*p*-value
Age (Years, $\bar{x}$ ± s)	55.07 ± 13.36	60.66 ± 12.27	8.60	<0.001
Male (%)	435 (61.18)	510 (58.22)	1.43	0.232
BMI (kg/m^2^, $\bar{x}$ ± s)	25.92 ± 3.32	22.98 ± 3.13	−18.12	<0.001
BMI			234.41	<0.001
Underweight, *N* (%)	1 (0.14)	48 (5.48)		
Normal weight, *N* (%)	213 (29.96)	527 (60.16)		
Overweight, *N* (%)	328 (46.13)	247 (28.20)		
Obesity, *N* (%)	169 (23.77)	54 (6.16)		
TC (mmol/L, $\bar{x}$ ± s)	4.51 ± 1.19	4.20 ± 1.14	−5.26	<0.001
TG (mmol/L, M (P25, P75))	1.45 (1.06, 2.13)	1.04 (0.78, 1.51)	−10.36	<0.001
HDL-C (mmol/L, $\bar{x}$ ± s)	1.04 ± 0.26	1.10 ± 0.33	3.48	<0.001
LDL-C (mmol/L, $\bar{x}$ ± s)	2.79 ± 0.87	2.52 ± 0.87	−6.05	<0.001
AST [IU/L, M (P25, P75)]	24.00 (19.00, 33.00)	20.00 (15.00, 24.00)	−11.01	<0.001
ALT [IU/L, M (P25, P75)]	27.00 (18.00, 41.00)	18.00 (13.00, 25.00)	−13.49	<0.001
γ-GGT [IU/L, M (P25, P75)]	33.00 (23.00, 59.75)	22.00 (15.00, 34.00)	−12.75	<0.001
HbA1c (%, $\bar{x}$ ± s)	9.32 ± 2.05	9.38 ± 2.52	0.43	0.664
FPG (mmol/L, $\bar{x}$ ± s)	8.86 ± 2.86	8.39 ± 3.33	−3.05	0.002
2 h PG (mmol/L, $\bar{x}$ ± s)	15.55 ± 4.16	16.68 ± 4.22	5.33	<0.001
FCP [ng/mL, M (P25, P75)]	1.69 (1.07, 2.54)	1.12 (0.64, 1.75)	−11.61	<0.001
2 h CP [ng/mL, M (P25, P75)]	6.10 (4.06, 9.25)	4.07 (2.40, 6.37)	−11.65	<0.001
IR indices				
HOMA-IR	0.63 (0.38, 0.97)	0.37 (0.21, 0.67)	−12.24	<0.001
QUICKI	0.20 (0.19, 0.21)	0.21 (0.20, 0.22)	−12.24	<0.001
IAI	0.07 (0.05, 0.12)	0.12 (0.07, 0.21)	−12.24	<0.001
Bennett ISI	0.40 (0.36, 0.45)	0.45 (0.38, 0.53)	−10.54	<0.001
ISI_matsuda_	1.14 (0.81, 1.71)	1.82 (1.14, 2.83)	−13.50	<0.001
ISI_0,120_	16.15 (14.18, 18.74)	16.82 (14.63, 19.86)	−3.35	<0.001

BMI, body mass index; TC, total cholesterol; TG, triglycerides; HDL-C, high-density lipoprotein cholesterol; LDL-C, low-density lipoprotein cholesterol; AST, aspartate aminotransferase; ALT, alanine aminotransferase; γ-GGT, gamma-glutamyl transpeptidase; HbAlc, glycosylated hemoglobin; FPG, fasting plasma glucose; 2 h PG, 2-h postprandial glucose; FCP, fasting C-peptide; 2 h CP, 2-h postprandial C-peptide; HOMA-IR, homeostatic model assessment of insulin resistance; QUICKI, quantitative insulin sensitivity check index; IAI, Li Guangwei index; Bennett ISI, Bennett insulin sensitivity index; ISI_matsuda_, Matsuda index; IS_I0,120_, Gutt index.

### 3.2 Comparison of clinical characteristics between T2DM patients with and without MASLD at different BMI levels

According to the BMI, T2DM patients were grouped into three categories: BMI < 24 kg/m^2^ (*n* = 789), 24 ≤ BMI < 28 kg/m^2^ (*n* = 575), and BMI ≥ 28 kg/m^2^ (*n* = 223). The differences in IR between the DM + MASLD and DM + Non-MASLD groups were compared at each BMI level. As shown in Supplementary Table S2, in the BMI < 24 kg/m^2^ and 24 ≤ BMI < 28 kg/m^2^, the DM + MASLD group exhibited higher HOMA-IR (0.51 vs. 0.32; 0.63 vs. 0.44) and lower values for QUICKI (0.21 vs. 0.22; 0.20 vs. 0.21), IAI (0.09 vs. 0.14; 0.07 vs. 0.10), Bennett ISI (0.17 vs. 0.19; 0.17 vs. 0.18), ISI_matsuda_ (1.32 vs. 2.08; 1.15 vs. 1.52), and ISI_0,120_ (16.09 vs. 16.88; 16.09 vs. 16.69) (all *p* < 0.05). In the BMI ≥ 28 kg/m^2^, the DM + MASLD group showed a significantly lower ISI_matsuda_ (0.91 vs. 1.12, *p* = 0.011), while no significant differences were found in other IR indices (*p* > 0.05).

### 3.3 Comparison of clinical characteristics among T2DM patients with MASLD at different liver fibrosis risk groups

Liver fibrosis risk stratification was performed in the DM + MASLD population according to the FIB-4 index: low risk group (*n* = 394), moderate risk group (*n* = 271), and high risk group (*n* = 46). Supplementary Table S3 shows that compared with the low- and moderate-risk groups, the high-risk group had significantly elevated levels of age (65.91 vs. 48.72; 65.91 vs. 62.46), AST (44 vs. 22; 44 vs. 26), ALT (35 vs. 28; 35 vs. 25), and *γ*-GGT (62 vs. 33; 62 vs. 31) (all *p* < 0.01). There were no significant differences observed among the three groups for BMI, HbA1c, FCP, 2 h PG, 2 h CP, or the prevalence of overweight/obesity (all *p* > 0.05). Furthermore, no significant differences in HOMA-IR, QUICKI, IAI, Bennett ISI, ISI_matsuda_, or ISI_0,120_ were observed across the different fibrosis risk groups (all *p* > 0.05).

### 3.4 Detection rates of MASLD and liver fibrosis at different IR levels

The participants were divided into four groups (Q1, Q2, Q3, Q4) based on the quartiles of HOMA-IR, QUICKI, IAI, Bennett ISI, ISI_matsuda_, and ISI_0,120_, respectively.

**Figure 2:**
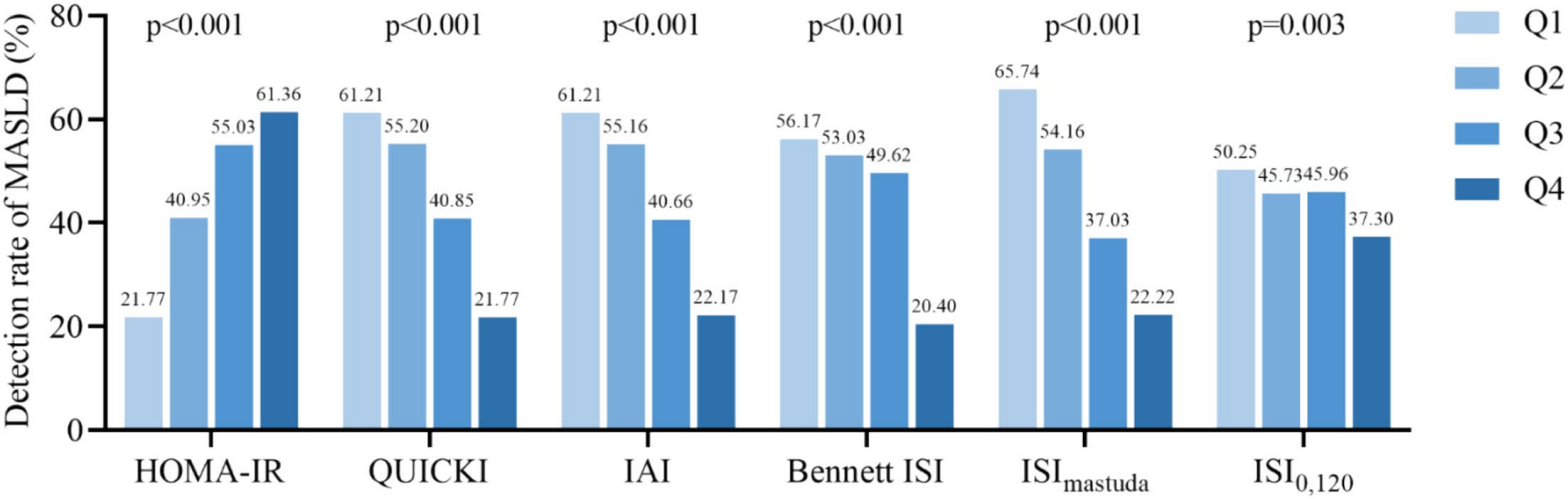
Detection rate of MASLD according to the quartiles of IR indicators. HOMA-IR, homeostatic model assessment of insulin resistance; QUICKI, quantitative insulin sensitivity check index; IAI, Li Guangwei index; Bennett ISI, Bennett insulin sensitivity index; ISI_matsuda_, Matsuda index; IS_I0,120_, Gutt index.

The findings indicated that for the basal IR indices, the MASLD detection rates for HOMA-IR Q1-Q4 were 21.77, 40.95, 55.03, and 61.36%, respectively. Conversely, the detection rates for QUICKI, IAI, and Bennett ISI decreased progressively across their quartile groups (all *p* < 0.001). Regarding post-stimulation IR indices, the MASLD detection rates for ISI_matsuda_ Q1–Q4 were 65.74, 54.16, 37.03, and 22.22%, respectively (*p* < 0.001). The MASLD detection rates for ISI_0,120_ Q1–Q4 were 50.25, 45.73, 45.96, and 37.30%, respectively (*p* = 0.003) (Figure 2).

For T2DM patients with MASLD, there were no significant differences in the detection rates of liver fibrosis across the quartile groups of HOMA-IR, QUICKI, IAI, Bennett ISI, ISI_matsuda_, and ISI_0,120_ (all *p* > 0.05) (Supplementary Figure S1).

### 3.5 Relationship between IR indices and the risk of MASLD and liver fibrosis in T2DM patients

**Figure 3:**
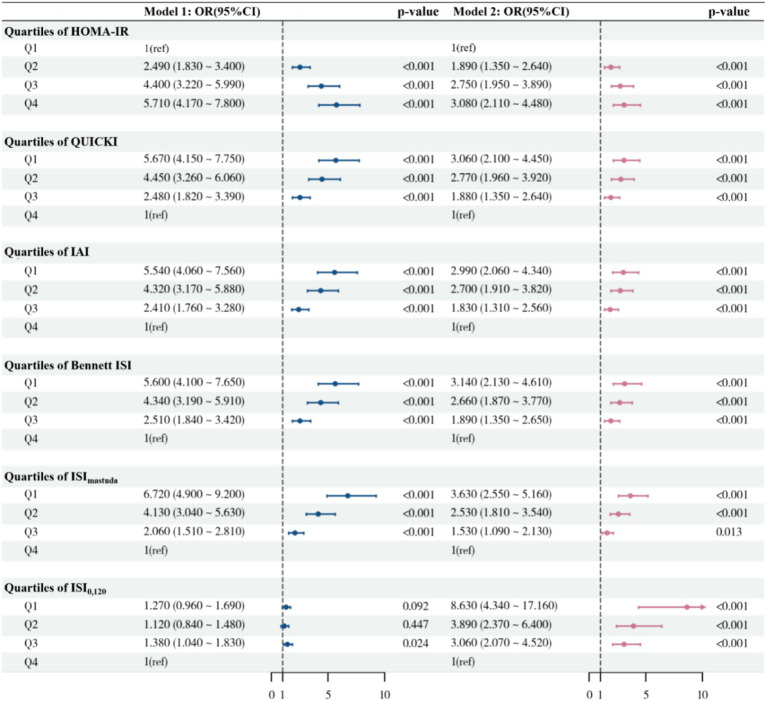
Logistic regression model for the association between IR indices and the risk of MASLD. Model 1, unadjusted; Model 2, adjusted for gender, age, BMI, FPG, 2 h PG. OR, odd ratio; 95 %CI, confidence interval; Ref, reference; HOMA-IR, homeostatic model assessment of insulin resistance; QUICKI, quantitative insulin sensitivity check index; IAI, Li Guangwei index; Bennett ISI, Bennett insulin sensitivity index; ISI_matsuda_, Matsuda index; IS_I0,120_, Gutt index.

Logistic regression analysis was conducted with MASLD as the dependent variable, and HOMA-IR, QUICKI, IAI, Bennett ISI, ISI_matsuda_, and ISI_0,120_ were each included separately as independent variables. Multicollinearity was assessed using VIF, and all covariates had VIFs <3, indicating no significant multicollinearity (Supplementary Table S4). In Model 2, after adjusting for gender, age, BMI, FPG, and 2 h PG, for the basal IR indices, the risks of MASLD in the Q2–Q4 groups of HOMA-IR, compared with the Q1 group, were 1.89 (95% CI: 1.35–2.64), 2.75 (95% CI: 1.95–3.89), and 3.08 (95% CI: 2.11–4.48), respectively. QUICKI, with the Q4 group as reference, the Q1–Q3 groups’ risks of MASLD were 3.06 (95% CI: 2.10–4.45), 2.77 (95% CI: 1.96–3.92), and 1.88 (95% CI: 1.35–2.64), respectively. Similar results were observed for IAI and Bennett ISI (all *p* < 0.001). For post-stimulation IR indices, compared with the Q4 group, the risks of MASLD for the Q1–Q3 groups for ISI_matsuda_ were 3.63 (95% CI: 2.55–5.16), 2.53 (95% CI: 1.81–3.54), and 1.53 (95% CI: 1.09–2.13), respectively, and for ISI_0,120_ they were 8.63 (95% CI: 4.34–17.16), 3.89 (95% CI: 2.37–6.40), and 3.06 (95% CI: 2.07–4.52) (all *p* < 0.001) (Figure 3). Similar results were obtained in Model 3, which additionally adjusted for gender, age, BMI, FPG, 2 h PG, TC, TG, HDL-C, LDL-C, UA, and hypertension status (Supplementary Table S5).

In the DM + MASLD group, with liver fibrosis as the dependent variable and HOMA-IR, QUICKI, IAI, Bennett ISI, ISI_matsuda_, and ISI_0,120_ as independent variables, logistic regression analysis indicated no significant differences in the risk of liver fibrosis across different indicator groups (all *p* > 0.05) (Supplementary Figure S2).

### 3.6 Predictive power of IR indices for MASLD in patients with T2DM

**Figure 4:**
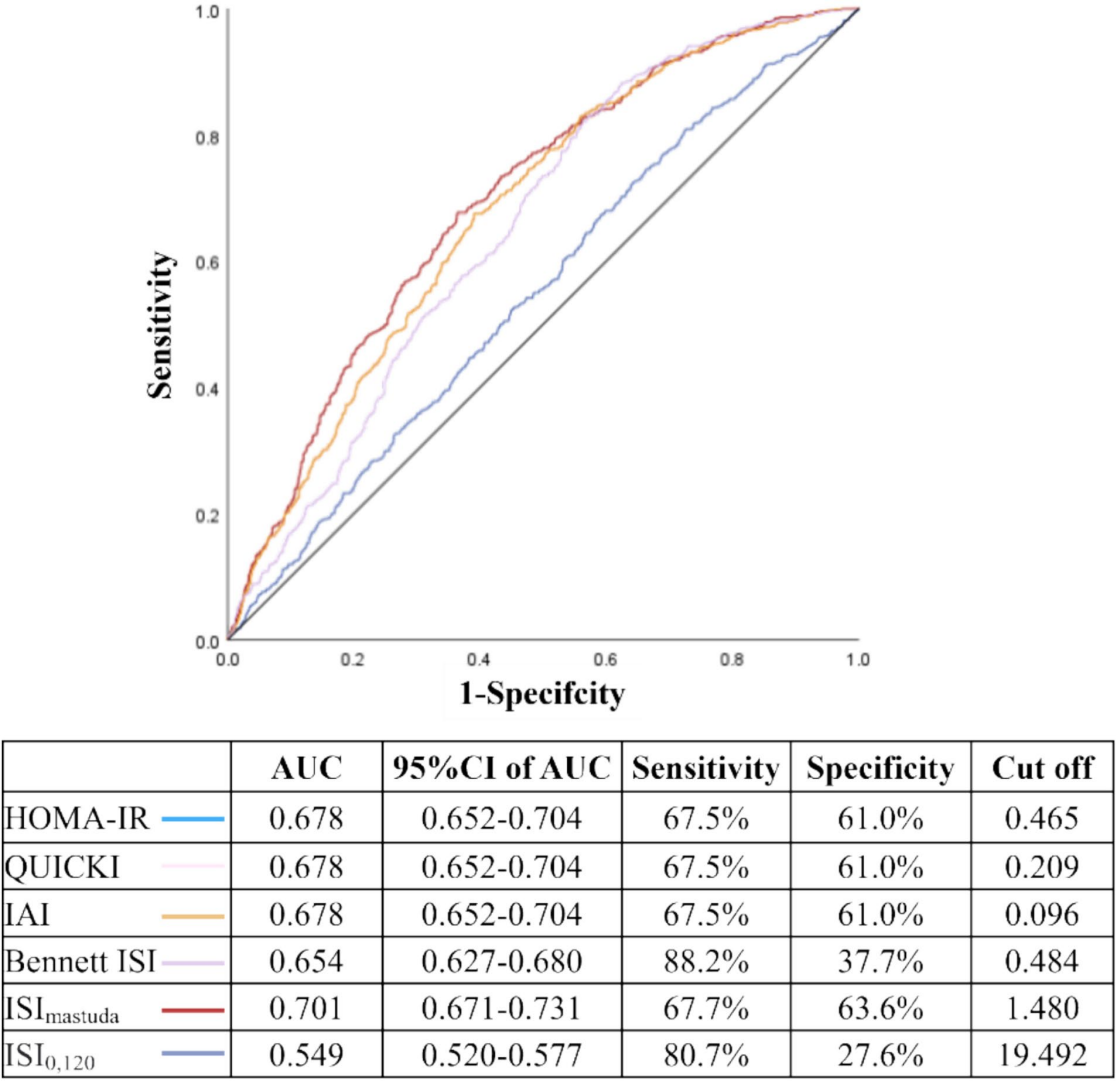
Predictive value of IR indices for MASLD in patients with T2DM. AUC: area under the curve; 95 %CI, confidence interval; HOMA-IR, homeostatic model assessment of insulin resistance; QUICKI, quantitative insulin sensitivity check index; IAI, Li Guangwei index; Bennett ISI, Bennett insulin sensitivity index; ISI_matsuda_, Matsuda index; IS_I0,120_, Gutt index. The ROC curves of HOMA-IR, QUICKI, and IAI completely overlap due to their mathematical monotonic relationships; thus, only a single curve is visually displayed for these three indices in this figure, although all were included in the analysis.

ROC curve analysis showed that the areas under the curves (AUC) for HOMA-IR, QUICKI, IAI, Bennett ISI, ISI_matsuda_, and ISI_0,120_ were 0.678, 0.678, 0.678, 0.654, 0.701, and 0.549, respectively. Notably, ISI_matsuda_ demonstrated the highest AUC at 0.701, with an optimal cutoff value of 1.480. The sensitivity was 67.7% and the specificity was 63.6% (Figure 4).

### 3.7 Development of a risk prediction model for MASLD in the T2DM population

We utilized clinically readily accessible indices—age, BMI, ALT, and TG—integrated into a logistic regression to develop a MASLD risk prediction model for the T2DM population. Based on our prior findings, the relationship between post-stimulation IR and MASLD was pronounced. Upon integrating 2 h CP into the MASLD risk prediction model, the C-index increased from 0.78 to 0.79. This enhancement substantially improved the model’s discriminatory power and risk reclassification (IDI 0.01, 95% CI: 0.00–0.02, *p* < 0.001; NRI 0.17, 95% CI: 0.06–0.29, *p* = 0.003), as shown in Supplementary Figure S3.

**Figure 5:**
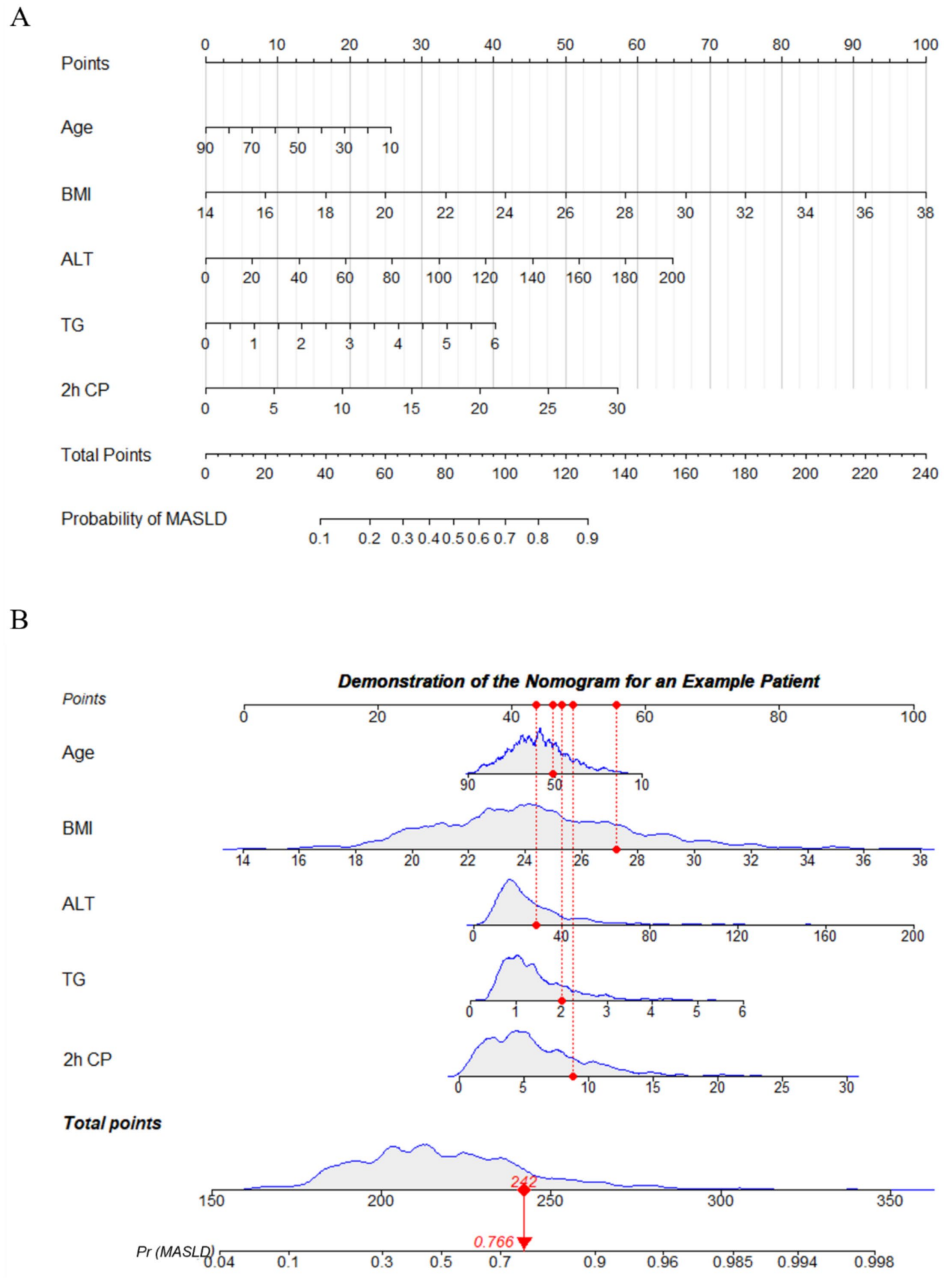
Nomogram to predict the risk of MASLD in diabetic patient. **(A)** To use the nomogram, the patient’s value for each variable (age, BMI, ALT, TG, 2 h CP) is located on its respective axis, and a vertical line is drawn upward to the “Points” axis to determine the corresponding score. The points for all variables are then summed to obtain the total score. Next, on the “Total Points” axis, the corresponding total is located, and a vertical line is drawn downward to the “Probability of MASLD” axis to obtain the patient’s predicted risk of MASLD. **(B)** Demonstration of nomogram use for an example patient. BMI, body mass index; ALT, alanine aminotransferase; TG, triglycerides; 2 h CP, 2-h postprandial C-peptide.

A nomogram was constructed based on age, BMI, ALT, TG, and 2 h CP to predict the probability of MASLD occurrence in patients with diabetes. This nomogram provided a visual method to calculate the total score based on the values of each predictor, offering an intuitive estimate of the likelihood of MASLD (Figure 5A). For example, as shown in Figure 5B, a T2DM patient aged 51 years with a BMI of 27.2 kg/m^2^, ALT of 28 IU/L, TG of 2 mmol/L, and 2 h CP of 8.80 ng/mL, the estimated probability of MASLD was 76.6%.

## 4 Discussion

Previous studies have demonstrated a close association between IR and the occurrence of MASLD, as well as the severity of liver involvement. In a small sample study of 56 patients with T2DM, Gala et al. identified that HOMA-IR was independently associated with the development of MASLD (OR = 1.5, 95% CI: 1.03–2.1, *p* = 0.033) (16). Similarly, Khamseh et al. conducted an investigation with 644 adult patients with T2DM and demonstrated that HOMA-IR (AUC = 0.713), QUICKI (AUC = 0.713), and Bennett ISI (AUC = 0.714) could all predict the onset of MASLD (17). In alignment with these findings, our research also revealed that the basal IR indices, including HOMA-IR, QUICKI, IAI, and Bennett ISI, were strongly associated with the occurrence of MASLD and had diagnostic value for MASLD. Clinical studies have also confirmed that insulin-sensitizing agents, such as thiazolidinediones (TZDs), can ameliorate T2DM with MASLD (18).

In addition, our observations revealed that compared with basal IR markers, post-stimulation IR indices such as ISI_0,120_ (OR = 8.63, 95%CI:4.34–17.16) and ISI_matsuda_ (OR = 3.63, 95%CI: 2.55–5.16) had stronger associations with MASLD. In our study, the highest MASLD detection rate was observed in the high IR group (Q1), as assessed by ISI_matsuda_, reaching 65.74%; ROC curve analysis demonstrated that the AUC of ISI_matsuda_ was significantly higher than that of the other IR indices (*p* < 0.01; Supplementary Table S6), suggesting that ISI_matsuda_ provides stronger diagnostic performance for MASLD. It is noteworthy that although ISI_0,120_ exhibited the highest OR, its AUC was the lowest among all IR indices, indicating that a strong association does not necessarily translate into good discriminative ability. Therefore, clinical evaluation of IR indices should incorporate multiple metrics, including both OR and AUC, to comprehensively assess their diagnostic and predictive value. Previous studies have shown that ISI_matsuda_ was significantly correlated with “gold-standard” HEC data (*r* = 0.73, *p* < 0.0001), outperformed HOMA-IR, and was capable of assessing both hepatic and peripheral insulin sensitivity simultaneously (19). In small sample studies using liver biopsy as the “gold standard” for diagnosing fatty liver, Kato et al. confirmed that ISI_matsuda_ was significantly correlated with the degree of hepatic steatosis (*r* = −0.45, *p* < 0.001) (20). In a prospective study of 37 patients with fatty liver who underwent metabolic surgery, ISI_matsuda_ was found to be superior to HOMA-IR in predicting the regression of NASH post-surgery (AUC = 0.98), highlighting its central role in the reversal of NASH (21, 22). Furthermore, in a study of 141 patients with T2DM using the proton magnetic resonance spectroscopy ([^1^H]-MRS) technique to quantify liver fat content (LFC), Wang et al. found an inverse correlation between increased LFC and ISI_matsuda_ (*r* = −0.214, *p* < 0.001) (23). To date, no large-scale clinical study has systematically compared the performance of basal versus post-stimulation IR indices in the diagnosis of MASLD, especially among high-risk populations with T2DM. Our research, involving 1,587 patients with T2DM, is the first to confirm that ISI_matsuda_ shows the most robust correlation with MASLD among indices used to assess IR. This suggests that post-stimulation IR may play a more critical role in the pathogenesis of fatty liver compared to basal IR. The underlying mechanism may involve the exacerbation of IR following stimulation, where postprandial hyperglycemic and hyperlipidemic states cause a “second hit,” facilitating an increased influx of FFA into the liver and thus accelerating the formation of fatty liver.

Under chronic hyperinsulinemia, the PI3K–Akt–FoxO1 pathway responsible for suppressing hepatic glucose production (HGP) becomes desensitized first, whereas the SREBP-1c branch that promotes DNL remains relatively intact. As a result, postprandial and nocturnal insulin levels are often above the threshold required to inhibit gluconeogenesis, but precisely within the range that maximally stimulates lipogenesis. This leads to the simultaneous occurrence of persistent hepatic glucose overproduction and increased triglyceride synthesis (24). This so-called “insulin window” mechanism is highly consistent with our clinical findings, which showed that lower ISI_matsuda_ values were associated with increased MASLD risk. In addition, the heterogeneity of hepatic metabolic zonation further exacerbates this metabolic imbalance. Studies using Gls2^CreER^ (periportal, PP) and Cyp1a2^CreER^ (pericentral, PC) insulin signaling knockout mouse models have demonstrated that PP hepatocytes predominantly mediate the inhibitory effect of insulin on HGP. Selective IR in the PP region alone can lead to impaired glucose tolerance and a complete loss of insulin-mediated regulation of HGP. However, DNL is jointly driven by both PP and PC regions, and disruption of insulin signaling in either region can reduce overall hepatic DNL levels and attenuate the risk of high-fat-diet–induced hepatic steatosis (25). These findings underscore the importance of dynamic assessment of postprandial IR and the implementation of targeted intervention strategies in the management of patients with T2DM. Evaluating IR solely by fasting insulin measurement may underestimate the true degree of IR in patients with diabetes and concomitant fatty liver. For patients with IR and fatty liver, choosing medications that can improve IR, such as TZDs, SGLT-2 inhibitors, or GLP-1 receptor agonists, may provide greater clinical benefits. Notably, in a phase II clinical trial, nearly 60% of NASH patients achieved histological remission after 48 weeks of semaglutide treatment, further highlighting its translational potential (26).

Currently, diagnostic models for fatty liver disease are predominantly developed for the general population, with a significant paucity of large-scale studies concentrating specifically on patients with T2DM. For instance, Cen et al. conducted a cross-sectional study involving 21,468 Chinese individuals, employing clinical indices such as BMI, diastolic blood pressure (DBP), UA, FPG, TG, and ALT to construct a clinical diagnostic model for MASLD, achieving an AUC of 0.857 (27). Katarzyna and colleagues, in their investigation of 1,735 diabetic patients, utilized eight clinical indices—age, BMI, type of diabetes, ALT, AST, hyperuricemia, platelet count, and metformin treatment—combined with a machine-learning approach, successfully developed an identification model for MASLD (AUC = 0.84) (28). Using simple clinical indices to predict the likelihood of fatty liver in diabetic populations can effectively conserve clinical resources. We selected age, BMI, TG, and ALT. Additionally, due to the complex calculation of ISI_matsuda_, we further incorporated the postprandial 2 h CP to establish a diagnostic model for MASLD in the population with T2DM, and found that adding 2 h CP markedly improved the model’s performance. Based on these indices, we constructed a nomogram to predict the probability of MASLD occurrence. Unlike previous research, our model simplifies the indices and, for the first time, includes postprandial 2 h CP as a critical predictive marker in MASLD diagnosis, highlighting the pivotal role of post-stimulation insulin levels in the development of MASLD.

The existing literature investigating the relationship between IR and the progression of liver fibrosis in patients with MASLD is limited. A longitudinal prospective cohort study conducted in South Korea, which included 10,030 adults, demonstrated that although the Homeostatic Model Assessment of HOMA-IR could predict the onset of fatty liver, it did not show a significant correlation with advanced liver fibrosis, defined as an FIB-4 score of 2.67 or higher (29, 30). Conversely, studies involving patients with biopsy-confirmed fatty liver, such as the work by Fujii et al., suggested that HOMA-IR (OR = 2.85, 95% CI: 1.21–7.19; *p* = 0.016) served as an independent risk factor for progressive liver fibrosis (31). Thus, the relationship between IR and liver fibrosis in MASLD patients remains contentious within the general population. Furthermore, there is a notable scarcity of related research specifically addressing individuals with T2DM. A study involving 483 patients with T2DM found no significant association between HOMA-IR and the presence of liver fibrosis, defined by a Liver Stiffness Measurement (LSM) of ≥8.0 kPa (32). Similarly, research conducted by Seeberg et al. on severely obese patients with T2DM (BMI ≥ 33 kg/m^2^) revealed no notable correlation between HOMA-IR, ISI_matsuda_, and levels of liver fibrosis (33). These findings align with the conclusions drawn from our study. To elucidate the relationship between IR and the progression to liver fibrosis in patients with T2DM comorbidity with MASLD, further investigation through large-scale, prospective cohort studies is warranted.

In conclusion, this study demonstrates that IR is an independent risk factor for the development of MASLD in patients with T2DM. Importantly, our research is the first to establish that post-stimulus IR indices, specifically ISI_matsuda_ and ISI_0,120_, are more strongly associated with MASLD than basal indices. Nonetheless, no clear relationship is identified between IR indices and the progression of liver fibrosis in T2DM patients with fatty liver.

## 5 Limitation

This study explored the correlation between commonly utilized IR indices and the progression of MASLD and liver fibrosis in patients with T2DM. However, several limitations should be acknowledged. The cross-sectional design precludes the establishment of a causal relationship between IR and MASLD in patients with T2DM. Additionally, the study participants were exclusively hospitalized patients with T2DM from a tertiary hospital in eastern Zhejiang Province. Although there were no explicit geographical restrictions during recruitment, the single-center nature of this study limits the external generalizability of our findings, necessitating further validation in multicenter, geographically diverse prospective cohorts. Furthermore, MASLD diagnosis was based on abdominal ultrasonography, and liver fibrosis was assessed using the FIB-4 index; although these approaches are consistent with current clinical guidelines and appropriate for large-scale retrospective analyses, they may underestimate mild steatosis or early-stage fibrosis. Future studies should therefore integrate MRI-based proton density fat fraction (MRI-PDFF) and selective liver biopsy for more accurate validation. Finally, given the strictly retrospective nature of this analysis, detailed data on dietary habits, physical activity, and specific medication use were unavailable from existing medical records, limiting deeper exploration of the relationship between IR and MASLD. Prospective studies systematically incorporating these variables are essential to enhance study rigor and strengthen the reliability of conclusions.

## Data Availability

The data analyzed in this study is subject to the following licenses/restrictions: In view of privacy and data security concerns, the complete raw data cannot be publicly disclosed; however, they are available from the corresponding author upon reasonable request. Requests to access these datasets should be directed to zhangmc1015@wmu.edu.cn.
