# Supplementary material for: ISImatsuda as a potential predictor of metabolic dysfunction-associated steatotic liver disease in patients with type 2 diabetes mellitus
Source: Front Med (Lausanne). 2025 Aug 29;12:1623808. doi: 10.3389/fmed.2025.1623808 (PMC12426025; doi:10.3389/fmed.2025.1623808)
Supplement: Supplementary file 1 [file Data_Sheet_1.docx]

**Table S1** Definition of indicators of insulin resistance.

| **Variables** | **Formulas** |
| --- | --- |
| HOMA-IR  QUICKI  IAI  Bennett ISI  ISI_mastuda_  ISI_0,120_ | FPG(mmol/L) ⅹ FCP(ng/mL) / 22.5  1 / (LogFPG(mg/dL) + LogFCP(pmol/L) )  1 / (FPG(mmol/L) × FCP(ng/mL) )  1/ (LogFPG(mmol/L) ×LogFCP(ng/mL) )  10000 / [ (FPG(mmol/L) × FCP(pmol/L) )^1/2^ ⅹ (G_mean_(mmol/L) ×C_mean_(pmol/L) )^1/2^]  75000 + (FPG(mmol/L)－2h PG(mmol/L))ⅹ0.19ⅹBW(kg) / 120 ⅹG_mean_(mmol/L) ×Log[C_mean_(pmol/L) ] |

Abbreviations: HOMA-IR, homeostatic model assessment of insulin resistance; QUICKI, quantitative insulin sensitivity check index; IAI, li guangwei index; Bennett ISI, bennett insulin sensitivity index; ISI_matsuda_, matsuda index; IS_I0,120_, gutt index; FPG, fasting plasma glucose; FCP, fasting C-peptide; 2h PG, 2-hour postprandial glucose; G_mean_, average blood glucose; C_mean_, average C-peptid.

**Table S2** Clinical characteristics of T2DM patients with and without MASLD at different BMI levels.

|  | **BMI < 24 kg/m^2^** | | | |  | **24 kg/m^2^ ≤ BMI < 28 kg/m^2^** | | | |  | **BMI ≥ 28 kg/m^2^** | | | |
| --- | --- | --- | --- | --- | --- | --- | --- | --- | --- | --- | --- | --- | --- | --- |
|  | DM+MAFLD | DM+Non-MAFLD | X^2^ /t/Z | P-Value |  | DM+MAFLD | DM+Non-MAFLD | X^2^ /t/Z | P-Value |  | DM+MAFLD | DM+Non-MAFLD | X^2^ /t/Z | P-Value |
| N (%) | 214(27.12) | 575(72.88) |  |  |  | 328(57.04) | 247(42.96) |  |  |  | 169(75.78) | 54(24.22) |  |  |
| HbA1c（%,‾x±s) | 9.27±2.01 | 9.66±2.64 | 1.770 | 0.078 |  | 9.36±2.12 | 8.95±2.28 | -1.810 | 0.071 |  | 9.30±2.00 | 8.70±1.93 | -1.68 | 0.095 |
| FPG (mmol/L,‾x±s) | 8.59±2.92 | 8.31±3.38 | -1.150 | 0.250 |  | 8.90±2.70 | 8.26±3.03 | -2.690 | 0.007 |  | 8.30(6.90,10.40) | 9.15(7.38,11.47) | -1.27 | 0.204 |
| 2h PG (mmol/L,‾x±s) | 16.25±4.10 | 17.10±4.30 | 2.500 | 0.012 |  | 15.66±4.15 | 15.91±3.85 | 0.740 | 0.457 |  | 14.46±4.06 | 15.73±4.45 | 1.95 | 0.053 |
| FCP (ng/Ml, M(P25,P75)) | 1.39(0.87,2.07) | 0.97(0.52,1.56) | -6.190 | <0.001 |  | 1.67(1.08,2.52) | 1.32(0.89,2.07) | -3.830 | <0.001 |  | 2.13(1.46,3.18) | 1.72(1.15,2.38) | -2.6 | 0.009 |
| 2h CP (ng/Ml, M(P25,P75)) | 5.26(3.81,8.12) | 3.73(2.08,5.68) | -7.650 | <0.001 |  | 6.40(4.00,9.03) | 5.12(3.09,8.31) | -3.060 | 0.002 |  | 7.02(4.83,11.15) | 5.26(3.51,7.89) | -3.41 | <0.001 |
| IR indices |  |  |  |  |  |  |  |  |  |  |  |  |  |  |
| HOMA-IR | 0.51(0.31,0.78) | 0.32(0.17,0.57) | -6.43 | <0.001 |  | 0.63(0.39,0.99) | 0.44(0.30,0.79) | -4.89 | <0.001 |  | 0.83(0.57,1.17) | 0.69(0.52,1.00) | -1.81 | 0.070 |
| QUICKI | 0.21(0.20,0.22) | 0.22(0.20,0.23) | -6.43 | <0.001 |  | 0.20(0.20,0.21) | 0.21(0.20,0.22) | -4.89 | <0.001 |  | 0.20(0.19,0.20) | 0.20(0.19,0.21) | -1.81 | 0.070 |
| IAI | 0.09(0.06,0.14) | 0.14(0.08,0.26) | -6.43 | <0.001 |  | 0.07(0.04,0.11) | 0.10(0.06,0.15) | -4.89 | <0.001 |  | 0.05(0.04,0.08) | 0.06(0.04,0.09) | -1.81 | 0.070 |
| Bennett ISI | 0.17(0.16,0.19) | 0.19(0.17,0.21) | -6.50 | <0.001 |  | 0.17(0.16,0.18) | 0.18(0.16,0.19) | -5.04 | <0.001 |  | 0.16(0.15,0.17) | 0.17(0.15,0.17) | -1.49 | 0.135 |
| ISI_matsuda_ | 1.32(0.94,2.08) | 2.08(1.35,3.31) | -7.73 | <0.001 |  | 1.15(0.82,1.68) | 1.52(1.02,2.16) | -4.89 | <0.001 |  | 0.91(0.68,1.33) | 1.12(0.82,1.57) | -2.54 | 0.011 |
| ISI_0,120_ | 16.09(13.91,18.74) | 16.88(14.70,19.82) | -2.58 | 0.010 |  | 16.09(14.16,18.58) | 16.69(14.61,20.13) | -2.21 | 0.027 |  | 16.41(14.47,18.94) | 15.44(13.48,18.56) | -1.240 | 0.215 |

Abbreviations: HbAlc, glycosylated hemoglobin; FPG, fasting plasma glucose; 2h PG, 2-hour postprandial glucose; FCP, fasting C-peptide; 2h CP, 2-hour postprandial C-peptide; HOMA-IR, homeostatic model assessment of insulin resistance; QUICKI, quantitative insulin sensitivity check index; IAI, li guangwei index; Bennett ISI, bennett insulin sensitivity index; ISI_matsuda_, matsuda index; IS_I0,120_, gutt index.

**Table S3** Clinical characteristics of T2DM patients with MASLD among different liver fibrosis risk groups.

|  | **FIB-4<1.3** | **1.3≤FIB-4≤2.67** | **FIB-4>2.67** | **F** | **P-Value** |
| --- | --- | --- | --- | --- | --- |
| N (%) | 394(55.41) | 271(38.12) | 46(6.47) |  |  |
| Age (Years,‾x±s) | 48.72±12.38 | 62.46±9.55^a^ | 65.91±11.33^ab^ | 141.03 | <0.001 |
| Male(%) | 257(65.23) | 147(54.24)^a^ | 31(67.39) | 8.96 | 0.011 |
| BMI (kg/m^2^, M(P25,P75)) | 25.83(23.83,27.78) | 25.18(23.36,27.75) | 25.50(23.55,27.34) | 4.13 | 0.127 |
| TC (mmol/L,‾x±s) | 4.67±1.25 | 4.30±1.09^a^ | 4.51±1.01 | 7.86 | <0.001 |
| TG (mmol/L, M(P25,P75)) | 1.61(1.09,2.30) | 1.33(1.00,1.93)^a^ | 1.43(1.10,1.92)^a^ | 10.88 | 0.004 |
| HDL-C (mmol/L,‾x±s) | 1.03±0.27 | 1.05±0.24^a^ | 1.09±0.25^a^ | 1.19 | 0.304 |
| LDL-C (mmol/L,‾x±s) | 2.90±0.89 | 2.63±0.83^a^ | 2.77±0.76^a^ | 7.87 | <0.001 |
| AST (IU/L, M(P25,P75)) | 22.00(17.00,28.00) | 26.00(21.50,34.50)^a^ | 44.00(31.50,56.00)^ab^ | 92.11 | <0.001 |
| ALT (IU/L, M(P25,P75)) | 28.00(19.00,41.00) | 25.00(17.00,39.00) | 35.00(26.25,50.75)^ab^ | 9.72 | 0.008 |
| γ-GGT (IU/L, M(P25,P75)) | 33.00(23.00,60.00) | 31.00(21.00,49.75) | 62.00(33.50,127.00)^ab^ | 19.60 | <0.001 |
| HbA1c（%,‾x±s) | 9.47±1.99 | 9.18±2.01 | 9.02±2.61 | 1.33 | 0.266 |
| FPG (mmol/L,‾x±s) | 8.50(7.03,10.57) | 7.90(6.50,9.90)^a^ | 7.85(6.62,9.78)^a^ | 7.98 | 0.018 |
| 2h PG (mmol/L,‾x±s) | 15.24±4.21 | 15.99±4.14 | 15.60±3.68 | 2.66 | 0.071 |
| FCP (ng/Ml, M(P25,P75)) | 1.70(1.10,2.50) | 1.67(0.99,2.56) | 1.89(1.20,3.25) | 1.91 | 0.385 |
| 2h CP (ng/Ml, M(P25,P75)) | 6.21(4.06,9.30) | 5.84(4.08,8.82) | 6.94(3.96,10.19) | 0.81 | 0.668 |
| IR indices |  |  |  |  |  |
| HOMA-IR | 0.66(0.42,0.97) | 0.57(0.34,0.96) | 0.61(0.44,1.01) | 3.97 | 0.138 |
| QUICKI | 0.20(0.19,0.21) | 0.20(0.20,0.21) | 0.20(0.19,0.21) | 3.97 | 0.138 |
| IAI | 0.07(0.05,0.11) | 0.08(0.05,0.13) | 0.07(0.04,0.10) | 3.97 | 0.138 |
| Bennett ISI | 0.39(0.36,0.44) | 0.41(0.36,0.46) | 0.40(0.36,0.44) | 4.46 | 0.107 |
| ISI_matsuda_ | 1.11(0.81,1.65) | 1.19(0.81,1.86) | 1.08(0.68,1.66) | 2.05 | 0.359 |
| ISI_0,120_ | 16.23(14.31,18.88) | 15.99(14.05,18.61) | 16.26(13.79,19.21) | 0.65 | 0.722 |

Abbreviations: FIB-4, fibrosis 4 index; BMI, body mass index; TC, total cholesterol; TG, triglycerides; HDL-C, high-density lipoprotein cholesterol; LDL-C, low-density lipoprotein cholesterol; AST, aspartate aminotransferase; ALT, alanine aminotransferase; γ-GGT, gamma-glutamyl transpeptidase; HbAlc, glycosylated hemoglobin; FPG, fasting plasma glucose; 2h PG, 2-hour postprandial glucose; FCP, fasting C-peptide; 2h CP, 2-hour postprandial C-peptide; HOMA-IR, homeostatic model assessment of insulin resistance; QUICKI, quantitative insulin sensitivity check index; IAI, li guangwei index; Bennett ISI, bennett insulin sensitivity index; ISI_matsuda_, matsuda index; IS_I0,120_, gutt index.

^a^ *P* <0.05 vs. FIB-4＜1.3, ^b^ *P* <0.05 vs. 1.3≤FIB-4≤2.67.

**
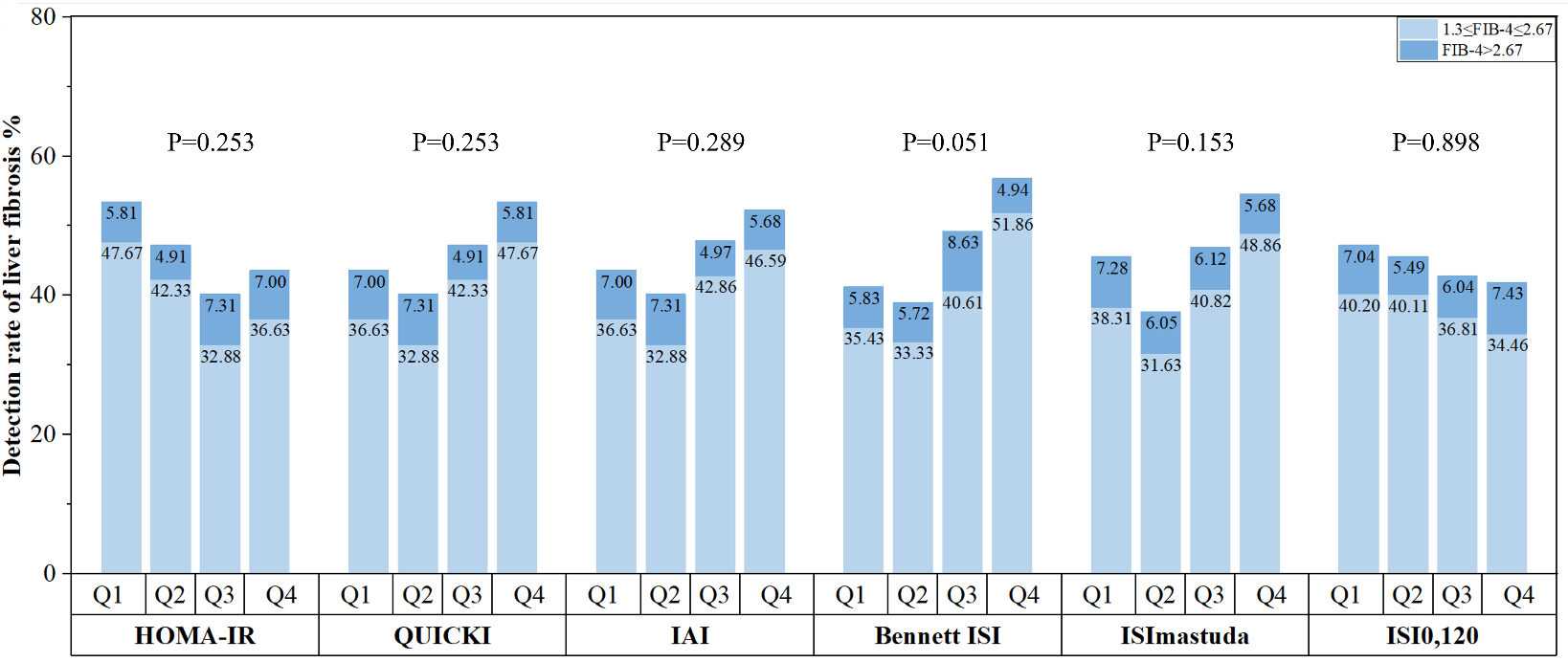
Figure S1.** Detection rate of liver fibrosis according to the quartiles of IR indicators in DM+MASLD patients.

Abbreviations: FIB-4, fibrosis 4 index; HOMA-IR, homeostatic model assessment of insulin resistance; QUICKI, quantitative insulin sensitivity check index; IAI, li guangwei index; Bennett ISI, bennett insulin sensitivity index; ISI_matsuda_, matsuda index; IS_I0,120_, gutt index.

**Table S4** VIF detection in logistic regression.

| Variables | Age | Gender | BMI | FPG | 2h PG | HOMA-IR |
| --- | --- | --- | --- | --- | --- | --- |
| VIF | 1.12 | 1.03 | 1.18 | 1.17 | 1.13 | 1.25 |
| Variables | Age | Gender | BMI | FPG | 2h PG | QUICKI |
| VIF | 1.12 | 1.03 | 1.26 | 1.14 | 1.17 | 1.34 |
| Variables | Age | Gender | BMI | FPG | 2h PG | IAI |
| VIF | 1.13 | 1.03 | 1.13 | 1.04 | 1.12 | 1.06 |
| Variables | Age | Gender | BMI | FPG | 2h PG | Bennett ISI |
| VIF | 1.12 | 1.03 | 1.18 | 1.21 | 1.14 | 1.30 |
| Variables | Age | Gender | BMI | FPG | 2h PG | ISI_mastuda_ |
| VIF | 1.13 | 1.03 | 1.13 | 1.04 | 1.11 | 1.04 |
| Variables | Age | Gender | BMI | FPG | 2h PG | ISI_0,120_ |
| VIF | 1.12 | 1.03 | 1.18 | 1.08 | 2.25 | 2.22 |

**Table S5** Logistic regression model 3 for the association between IR indices and the risk of MASLD**.**

| Variables | OR | 95% CI | | P-Value |
| --- | --- | --- | --- | --- |
|  |  | Lower | Upper |  |
| Quartiles of HOMA-IR |  |  |  |  |
| Q1 | 1.00 (Ref) |  |  |  |
| Q2 | 1.82 | 1.21 | 2.73 | 0.004 |
| Q3 | 2.20 | 1.43 | 3.37 | <0.001 |
| Q4 | 2.58 | 1.57 | 4.23 | <0.001 |
| Quartiles of QUICKI |  |  |  |  |
| Q1 | 2.58 | 1.58 | 4.23 | <0.001 |
| Q2 | 2.21 | 1.44 | 3.39 | <0.001 |
| Q3 | 1.81 | 1.20 | 2.72 | 0.004 |
| Q4 | 1.00 (Ref) |  |  |  |
| Quartiles of IAI |  |  |  |  |
| Q1 | 2.48 | 1.52 | 4.06 | <0.001 |
| Q2 | 2.16 | 1.41 | 3.30 | <0.001 |
| Q3 | 1.76 | 1.17 | 2.65 | 0.006 |
| Q4 | 1.00 (Ref) |  |  |  |
| Quartiles of Bennett ISI |  |  |  |  |
| Q1 | 3.12 | 1.65 | 5.89 | <0.001 |
| Q2 | 2.80 | 1.73 | 4.54 | <0.001 |
| Q3 | 2.73 | 1.80 | 4.15 | <0.001 |
| Q4 | 1.00 (Ref) |  |  |  |
| Quartiles of ISI_mastuda_ |  |  |  |  |
| Q1 | 3.46 | 2.16 | 5.54 | <0.001 |
| Q2 | 2.17 | 1.43 | 3.30 | <0.001 |
| Q3 | 1.42 | 0.95 | 2.13 | 0.088 |
| Q4 | 1.00 (Ref) |  |  |  |
| Quartiles of ISI_0,120_ |  |  |  |  |
| Q1 | 6.62 | 3.08 | 14.25 | <0.001 |
| Q2 | 3.03 | 1.73 | 5.31 | <0.001 |
| Q3 | 2.35 | 1.51 | 3.66 | <0.001 |
| Q4 | 1.00 (Ref) |  |  |  |

Adjusted for gender ,age, BMI, FPG, 2h PG, TC, TG, HDL-C, LDL-C, UA , and hypertension status.

Abbreviations: OR, odd ratio; 95 %CI, confidence interval; Ref, reference; BMI, body mass index; FPG, fasting plasma glucose; 2h PG, 2-hour postprandial glucose; TC, total cholesterol; TG, triglycerides; HDL-C, high-density lipoprotein cholesterol; LDL-C, low-density lipoprotein cholesterol; UA, uric acid; HOMA-IR, homeostatic model assessment of insulin resistance; QUICKI, quantitative insulin sensitivity check index; IAI, li guangwei index; Bennett ISI, bennett insulin sensitivity index; ISI_matsuda_, matsuda index; IS_I0,120_, gutt index.


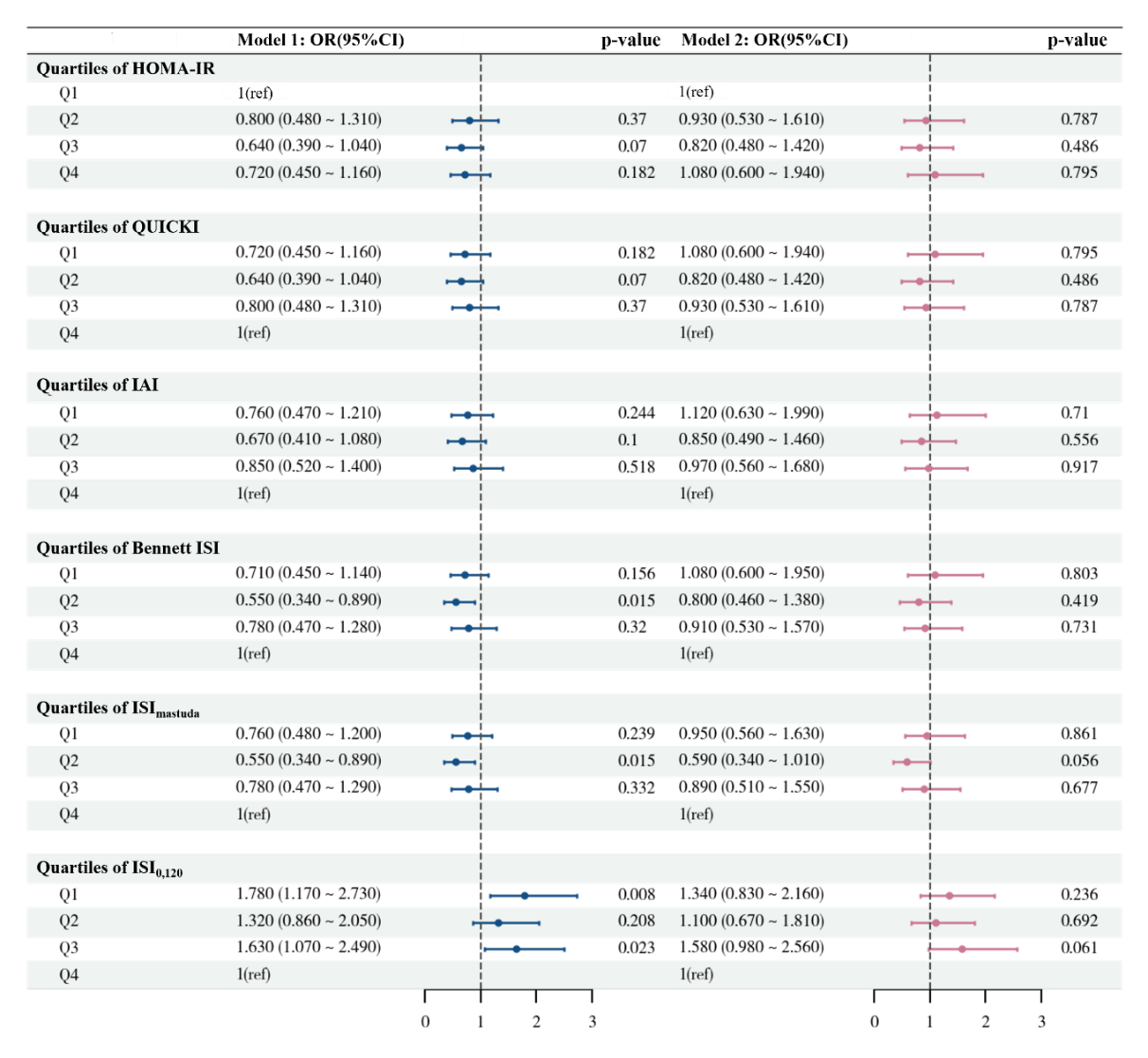


**Figure S2** Logistic regression models for the association between IR indices and the risk of liver fibrosis.

Model 1, unadjusted; Model 2, adjusted for gender ,age, BMI, FPG, 2h PG.

Abbreviations: OR, odd ratio; 95 %CI, confidence interval; Ref, reference; HOMA-IR, homeostatic model assessment of insulin resistance; QUICKI, quantitative insulin sensitivity check index; IAI, li guangwei index; Bennett ISI, bennett insulin sensitivity index; ISI_matsuda_, matsuda index; IS_I0,120_, gutt index.

**Table S6** Comparison of AUCs Between Insulin Resistance Indices for MASLD Prediction.

| Variables | Difference between areas | Standard Error | 95% CI | z | p-value |
| --- | --- | --- | --- | --- | --- |
|  |  |  |  |  |  |
| HOMA-IR vs ISI_matsuda_ | -0.023 | 0.161 | (-0.030, -0.007) | -3.09 | 0.002 |
| QUICKI vs ISI_matsuda_ | -0.023 | 0.161 | (-0.030, -0.007) | -3.09 | 0.002 |
| IAI vs ISI_matsuda_ | -0.023 | 0.161 | (-0.030, -0.007) | -3.09 | 0.002 |
| Bennett ISI vs ISI_matsuda_ | -0.047 | 0.162 | (-0.064, -0.023) | -4.11 | <0.001 |
| ISI_matsuda_ vs ISI_0,120_ | 0.152 | 0.165 | (0.119, 0.177) | 10.044 | <0.001 |

Abbreviations: 95 %CI, confidence interval; HOMA-IR, homeostatic model assessment of insulin resistance; QUICKI, quantitative insulin sensitivity check index; IAI, li guangwei index; Bennett ISI, bennett insulin sensitivity index; ISI_matsuda_, matsuda index; IS_I0,120_, gutt index.

**
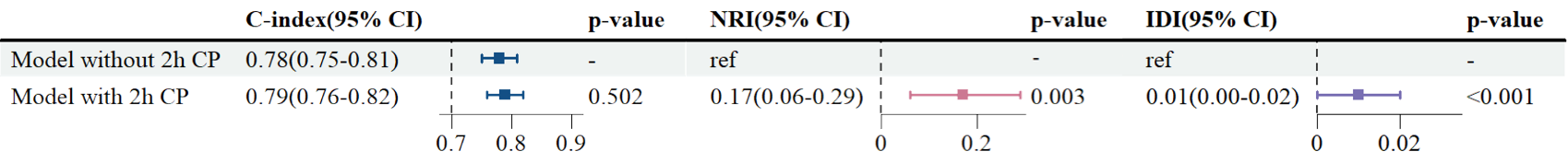
**

**Figure S3** Evaluation of modeling improvement with and without 2h CP for diagnostic prediction of MASLD.

Model without 2h CP, including Age, BMI, ALT, TG.

Abbreviations: 2h CP, 2-hour postprandial C-peptide; C index, Harrell’s concordance statistic; NRI, net reclassification improvement; IDI, integrated discrimination improvement.
